# Sleep impact of pandemic COVID-19 crisis on university students in Saudi Arabia and associated factors

**DOI:** 10.1192/j.eurpsy.2022.2097

**Published:** 2022-09-01

**Authors:** A. Alhadi, A. Alhuwaydi

**Affiliations:** 1 King Saud University, Sabic Psychological Health Research & Applications Chair, Department Of Psychiatry, College Of Medicine, Riyadh, Saudi Arabia; 2 Jouf University, Department Of Medicine, College Of Medicine, Sakaka, Saudi Arabia

**Keywords:** Covid-19, Insomnia, University student, Saudi Arabia

## Abstract

**Introduction:**

COVID-19 pandemic has many psychological and physical effects. University students are among vulnerable population.

**Objectives:**

We aimed in this study to assess sleep effects of COVID-19 pandemic on university students in Saudi Arabia.

**Methods:**

We conducted cross-sectional study to collect responses of 5,140 participations from Saudi universities, responders completed the demographic questions, psychological scales including insomnia severity scale (ISI) between 24th and 30th of April 2020.

**Results:**

About 41% of the sample suffered from moderate to severe insomnia. Mean ISI score was 12.9 (SD 6.62). Insomnia was associated with female gender, younger age, students from new universities, junior students, if a relative got COVID-19, having a chronic medical illness, and having a psychiatric disorder.

**Conclusions:**

Covid-19 pandemic has clear effect on sleep among Saudi university students. Universities need to plan and implement protective and intervention strategies to deal with this important issue.

**Disclosure:**

No significant relationships.

